# Cell wall modifications that alter the exolytic activity of lactococcal phage endolysins have little impact on phage growth

**DOI:** 10.3389/fmicb.2023.1106049

**Published:** 2023-01-20

**Authors:** Susana Escobedo, Mikel Pérez de Pipaon, Claudia Rendueles, Ana Rodríguez, Beatriz Martínez

**Affiliations:** Instituto de Productos Lacteos de Asturias (IPLA), CSIC, Villaviciosa, Spain

**Keywords:** bacteriophage, endolysins, *Lactococcus*, peptidoglycan, cell envelope stress

## Abstract

Bacteriophages are a nuisance in the production of fermented dairy products driven by starter bacteria and strategies to reduce the risk of phage infection are permanently sought. Bearing in mind that the bacterial cell wall plays a pivotal role in host recognition and lysis, our goal was to elucidate to which extent modifications in the cell wall may alter endolysin activity and influence the outcome of phage infection in *Lactococcus*. Three lactococcal endolysins with distinct catalytic domains (CHAP, amidase and lysozyme) from phages 1,358, p2 and c2 respectively, were purified and their exolytic activity was tested against lactococcal mutants either overexpressing or lacking genes involved in the cell envelope stress (CES) response or in modifying peptidoglycan (PG) composition. After recombinant production in *E. coli*, Lys1358 (CHAP) and LysC2 (muramidase) were able to lyse lactococcal cells in turbidity reduction assays, but no activity of LysP2 was detected. The degree of PG acetylation, namely C_6_-*O*-acetylation and de-*N*-acetylation influenced the exolytic activity, being LysC2 more active against cells depleted of the PG deacetylase PgdA and the *O-*acetyl transferase OatA. On the contrary, both endolysins showed reduced activity on cells with an induced CES response. By measuring several growth parameters of phage c2 on these lactococcal mutants (lytic score, efficiency of plaquing, plaque size and one-step curves), a direct link between the exolytic activity of its endolysin and phage performance could not be stablished.

## Introduction

Bacteriophages or phages are viruses that infect bacteria, they are ubiquitous and can be found in every ecosystem where bacteria exist. If present in the dairy factory environment, phage infection of lactic acid bacteria (LAB), specifically that of *Lactococcus lactis* and *Lactococcus cremoris* used in starter cultures, may interfere with bacterial growth and led to failures in milk fermentation. In order to reduce the risk, several measures have been implemented including the search for phage-resistant starter bacteria (Marcó et al., 2012).

The bacterial cell wall (CW) plays a pivotal role in host:phage interactions in LAB and its composition and architecture may determine resistance to phage infection (reviewed by Chapot-Chartier and Kulakauskas, 2014; Mahony et al., 2017; Martínez et al., 2020). Indeed, three of the infection steps (adsorption, DNA delivery and host lysis), involve CW components. Surface structures, e.g., cell wall polysaccharides (CWPS), membrane proteins and (lipo-)teichoic acids, act as discriminating recognition sites for phages and are prone to be modified and confer phage resistance (Viscardi et al., 2003; Chapot-Chartier and Kulakauskas, 2014; Bertozzi Silva et al., 2016). Once positioned, phages must eject their DNA inside the bacterial cell with the help of virion-associated enzymes that degrade CW polymers locally, allowing tail penetration and DNA passage without lysing the cell (Moak and Molineux, 2004; Latka et al., 2017). Later in the lytic cycle after building new phage particles, host lysis relies on the activity of phage-encoded endolysins that hydrolyze the peptidoglycan (PG), the main CW component in Eubacteria, made of glycan chains of N-acetyl-muramic acid (NAM) and N-acetyl-glucosamine (NAG) cross-linked through short peptide chains.

Endolysins from phages infecting Gram-positive bacteria often have a modular structure that includes a N-terminal domain, that specifies the catalytic activity of the enzyme, and a CW binding domain involved in substrate binding (Schmelcher et al., 2012; Oliveira et al., 2013). A recent insight into the diversity of lactococcal phage endolysins have uncovered 11 types of these modular enzymes (Oechslin et al., 2022). Identified catalytic domains include muramidases (phage_lysozyme, Glycohydro_25), N-acetyl-muramyl-L-Ala-amidases (Amidase_2 domain), γ-D-Glu-L-Lys-endopeptidases (Amidase_5) and CHAP, with both cysteine-histidine-dependent amidohydrolase and/or endopeptidase activity (Oliveira et al., 2013).

As described for host autolysins, resistance to PG-degrading enzymes may occur through chemical modifications of the CW components (Grishin et al., 2020). In *Lactococcus*, PG modifications such as NAG de-*N*-acetylation by the deacetylase PgdA and amidation of D-Asp in the PG cross bridge protects PG from hydrolysis by the major autolysin AcmA (Meyrand et al., 2007; Veiga et al., 2009), while changes in the composition of the lipoteichoic acid decreased substrate binding (Steen et al., 2008). However, resistance to the exolytic activity of phage endolysins is scarcely documented and even less is known if there are consequences for phage growth.

Exposure to harsh conditions or CW damaging agents may also change, at least, transiently, the structure of major CW components through the activation of signal transduction systems, mostly two-component systems (TCSs), that activate defense mechanisms, collectively known as the cell envelope stress (CES) response (Jordan et al., 2008). In *Lactococcus cremoris* MG1363, inhibition of CW biosynthesis by the lactococcal bacteriocin Lcn972 and PG hydrolysis by lysozyme are known to activate the TCS CesSR (Martínez et al., 2007; Veiga et al., 2007). Of interest for this work, the CES response has been shown to be also activated upon bacteriophage infection (Fallico et al., 2011). A main effector of CesSR is SpxB which, through binding to the RNA polymerase RpoA, drives transcription of *oatA*, encoding the *O*-acetyl transferase OatA that leads to *O*-acetylation at the C6-hydroxyl of N-acetylmuramoyl residues (Veiga et al., 2007). This is a widespread PG modification that sterically inhibits lysozyme but also the exolytic activity of the *L. cremoris* prophage TP712 endolysin (Escobedo et al., 2019).

Hence, aware of the importance of the bacterial CW for phage infection as well as the regulatory mechanisms dedicated to preserve its integrity, along with its multiple modifications, the aim of this work was to identify modifications of the lactococcal CW that could alter the exolytic activity of phage endolysins. As described above, these modifications could be either caused by mutations in genes coding for PG modifying enzymes or in those involved in the lactococcal CES response. The consequences for phage infection were also evaluated using the virulent phage c2, in order to decipher to which extent a thwarted endolysin activity may or may not restrict phage growth. The results show that while the CES response and the degree of PG-acetylation may indeed compromise or enhanced the exolytic activity of two endolysins, the impact on phage growth is negligible.

## Materials and methods

### Bacterial strains, bacteriophages, and growth conditions

Bacterial strains used in this study are listed in Table 1. *L. cremoris* strains were grown in M17 medium (Formedium, UK) supplemented with 0.5% glucose (GM17) at 30°C. Chloramphenicol (5 μg/ml) and erythromycin (2.5 μg/ml) were added as needed. *Escherichia coli* was grown in Luria Bertani (LB) or Terrific broth (TB; Sambrook et al., 1989) at 37°C with shaking (250 rpm) in the presence of 30 μg/ml kanamycin if needed.

**Table 1:** Bacterial strains, plasmids, and bacteriophages used in this work.

Strain	Description^1^	Reference
*Lactococcus cremoris*		
NZ9000	MG1363 *pepN::nisRK*. Wild type, phage c2 host	Kuipers et al. (1998)
MG1363	Plasmid-free derivative of NCDO712. Wild type, phage c2 host	Gasson (1983)
spxB+	MG1363, multicopy *spxB* (VES3910). Increased PG *O*-acetylation. CmR, LysR	Veiga et al. (2007)
oatA-	MG1363 lacking a functional *oatA* (VES4289). Absence of PG *O*-acetylation. LysS	Veiga et al. (2007)
pgdA*-*	MG1363 lacking PG deacetylase *pgdA* (VES4534). Fully acetylated PG. EmR, LysS	Meyrand et al. (2007)
pgd*A*+	MG1363, multicopy PG deacetylase *pgdA* (VES3787). Increased PG de-*N*-acetylation. CmR, LysR	Meyrand et al. (2007)
cesSR-	NZ9000 derivative with chromosomal deletion of *cesS* and *cesR*.	Pinto et al. (2011)
cesSR+	NZ9000, multicopy *cesSR.* EmR	Pinto et al. (2011)
*Escherichia coli*		
DH10B	Cloning host	Invitrogen
BL21 (DE3)	Gene expression host. CmR	Novagen
Plasmids		
pET-29b(+)	Inducible *E. coli* expression vector. KanR	Novagen
pETLysP2	*E. coli* codon optimized *lysP2* cloned in pET29-b(+)	This work
pETLysC2	*E. coli* codon optimized *lysC2* cloned in pET29-b(+)	This work
pETLys1358	*E. coli* codon optimized *lys1358* cloned in pET29-b(+)	This work
Bacteriophages		
c2	Prolate-headed virulent lactococcal phage	Pillidge and Jarvis (1988)

^1^Cm, chloramphenicol; Em, erythromycin; Kan, kanamycin; Lys, lysozyme; R, resistant; S, sensitive.

To propagate phage c2, *L. cremoris* MG1363 was grown in GM17 with 10 mM Ca(NO_3_)_2_ and 10 mM MgS0_4_ until an OD_600_ of 0.4 was reached and then infected at a MOI of 0.01. Incubation proceeded until lysis had occurred (≈6 h). The culture was then centrifugated, the lysate filtered through a polyethersulfone filter (0.2 μm) and stored at 4°C. Plaque-forming units (pfu/ml) were determined by the double-layer agar assay. Inoculating the top agar (0.7%) with 100 μl of overnight cultures of the bacterial host and appropriate phage dilutions prepared in SM buffer (20 mg/ml Tris–HCL, 10 mg/ml MgSO_4_, 10 mg/ml CaCl_2_, 100 mg/ml NaCl, pH 7.5).

### Expression and purification of lactococcal endolysins

The genes encoding the lactococcal endolysins LysP2, LysC2 and Lys1358 (Table 2) were optimized for *E. coli* codon usage by Twist DNA codon optimization technology (Twist Bioscience, San Francisco, CA, USA). The DNA fragments were then cloned into the NdeI and XhoI restriction sites of the pET29b + vector, which introduces a C-terminal His6 tag to yield pETLysP2, pETLysC2 and pETLys1358 (Table 1). *Escherichia coli* BL21 (DE3) harboring these plasmids were grown in 0.5 L of LB (pETLysP2, pETLys1358) or TB media (pETLysC2) with 30 μg/ml kanamycin at 37°C to an OD_600_ of 0.5. Gene expression was induced by addition of 1 mM IPTG. Following 18 h of incubation at 12°C (pETLysC2) or 16°C (pETLysP2, pETLys1358) bacterial cells were collected and resuspended in lysis buffer (50 mM NaH_2_PO_4_, 300 mM NaCl, 10 mM imidazole, pH 8) supplemented with 1 mg/ml of lysozyme (Merck, Germany) and 10 μg/ml of DNAse (Sigma, Spain). The cell suspension was then disrupted by sonication, and after centrifugation (20,000× *g*, 30 min), the supernatant was passed through a Ni-NTA superflow column (Qiagen, Germany) and eluted in a 50 mM NaH_2_PO_4_, 300 mM NaCl, 250 mM imidazole (pH 8) buffer according to the manufacturer’s instructions. Due to low yields, LysC2 was further concentrated and the buffer replaced with 50 mM sodium phosphate buffer, 150 mM NaCl (pH 7.5) using an Amicon Ultra-15 3-kDa-molecular-weight-cutoff (MWCO) filter unit (Merck KGaA, Germany). The proteins were stored at −20°C after the addition of glycerol at 25%. Protein concentration was determined by the Bradford assay (Bio-Rad Laboratories, Hercules, CA, USA) using bovine serum albumin as standard.

**Table 2:** Biochemical properties of the recombinant lactococcal endolysins used in this work.

Endolysin	GenBank	CD^1^ (Pfam)	Activity	CBD^2^ (Pfam)	pI^3^	MW^3^ (kDa)
LysP2	ADC80094.1	Amidase_2 (PF01510)	Amidase	Not identified	6.16	29.4
LysC2	NP_043551.1	Phage_lysozyme (PF00959)	Muramidase	Not identified	8.65	26.4
Lys1358	ADD25719.1	CHAP (PF05257)	Amidase or endopeptidase	SH3_5 (PF08460)	9.59	26.7

^1^CD, catalytic domain; ^2^CBD, cell wall binding domain. ^1,2^Assignments according to Chapot-Chartier and Kulakauskas, 2014. ^3^ Isoelectric point (pI) and molecular weight (MW) according to https://web.expasy.org/protparam/.

### SDS-PAGE and Zymograms

Sodium dodecyl sulfate (SDS)-PAGE was performed in a BioRad Mini-Protean gel apparatus (BioRad) using 10% (w/v) polyacrylamide separating gels. As previously described for zymograms (Lepeuple et al., 1998), the polyacrylamide gels contained 0.2% (w/v) *Micrococcus luteus* ATCC 4698 (Sigma) or 0.4% (w/v) *L. cremoris* NZ9000 autoclaved cells. Gels were subsequently washed for 30 min in deionized H_2_O at room temperature and incubated overnight at 37°C in 5 mM Tris–HCl, pH 7.5, supplemented with 0.1% Triton X-100 as renaturing buffer. Zymograms were photographed without further staining and Precision Plus Protein™ All Blue Standards (BioRad) was used as a molecular weight marker.

### Turbidity reduction assays

Quantitative detection of endolysin activity was performed *via* turbidity reduction assays. Lactococcal cells used as substrate were grown in 30 ml GM17 until an OD_600_ 0.5. Then, they were centrifuged at 3,500× *g* for 15 min at 4°C, washed with 30 ml 50 mM sodium phosphate buffer, pH 7.5, adjusted to a final OD_600_ 1.5 in the same buffer, aliquoted and frozen at −20°C. In a 96-well plate, 100 μl of the cell suspension was mixed with one volume of LysC2 and Lys1358 to reach a final concentration of 4.5 and 0.5 μM, respectively. OD_600_ was measured in a microtiter plate reader (Tecan Trading AG) at 30°C every 5 min for 1 h. Activity was defined as the decrease of mOD per minute (mOD/min). Two to seven replicates were carried out.

### Phage infection assays

Lysis-in-broth assays were performed by infecting early exponentially growing cultures at OD_600_ of 0.2 with c2 in the presence of 10 mM Ca(NO_3_)_2_ and 10 mM MgS0_4_ at a MOI of 0.2. Lysis was monitored in a 96-well plate microtiter plate reader at 10 min intervals for 290 min post-infection. All assays were performed in triplicate. To analyze the results, the area under the curve (AUC) of the treated cultures and the uninfected control for the first 140 min was calculated as previously described (Xie et al., 2018). The lytic score was defined as 1-AUC _treated_/AUC _control_. For comparisons, the lytic scores on the wild type strains were taken as 100%. The growth rate of the non-infected cultures (μ, h^−1^) was calculated by linear regression of Ln(OD) plotted against time.

The efficiency of plaquing (EOP) of c2 on the lactococcal mutants was defined as the ratio between the average number of plaques on the mutant strain by the average number of plaques of the wild type (MG1363 or NZ9000). EOP determination was carried out in four independent experiments. The diameter of five lysis plaques from each biological replicate (*n* = 20) was measured with a digital caliper implemented in ImageJ (https://imagej.nih.gov/ij/).

### Phage adsorption

Adsorption of phage to host cells was performed as described (Madera et al., 2003). Phage c2 was added at a MOI of 0.001 to stationary-phase host cultures diluted to an OD_600_ of 0.8 in GM17 supplemented with 10 mM of Ca(NO_3_)_2_ and 10 mM MgSO_4_. Following a 10 min incubation at room temperature, the phage-host mixture was centrifuged for 5 min, and phage counts in the supernatant determined by standard double-layer agar assays. A sample without cells was equally treated to determine the initial phage titer. The percentage adsorption was calculated as (1-residual phage titer/initial phage titer) × 100. Experiments were carried out with two to five independent lactococcal cultures.

### One-step growth curves

One-step growth curves were performed as previously described (Moineau et al., 1993), with some modifications. Briefly, 2 ml of log-phase cells (OD_60_0 = 0.8) of each host was centrifuged at 8,000× *g* for 5 min at 4°C. The pellet was resuspended in 900 μl of GM17 with 10 mM of Ca(N0_3_)_2_/10 mM of MgSO_4_, and 100 μl of phage solution were added to achieve a final MOI of 0.01. The mixture was left at room temperature for 5 min and then washed twice to remove unabsorbed phages. The mixture was diluted 10^4^-fold and incubated at 30°C. Samples were withdrawn periodically over 50 min, diluted, and spotted on *L. cremoris* MG1363 lawn for phage counts. The burst size (pfu per infected cell) was calculated as phage titer at the end of the one-step growth curve minus the initial titer and divided by the initial titer. The latent period was defined at the starting of the exponential phase and the burst time as the time invested to complete one cycle (Chmielewska-Jeznach et al., 2020).

### Statistical analyses

A two-tailed Student t-test as implemented in Microsoft Excel 2019 was applied for comparison. A value of *p* threshold of 0.05 was set for significance.

## Results

### Recombinant production of lactococcal phage endolysins

The endolysins LysP2, LysC2 and Lys1358 with different catalytic domains (Table 2) were produced as C-terminally His-tagged proteins. The induction conditions, namely the growth medium and the temperature of incubation, were optimized for each endolysin. LysP2 and Lys1358 were successfully produced in the soluble fraction in LB at 16°C, yielding 3 and 16.5 mg per 0.5 L of induced *E. coli* cultures after purification, respectively. However, under these inducing conditions, LysC2 was insoluble but purification could be achieved after induction in TB and at lower T (12°C), reaching up to 3.3 mg per 0.5 L of induced cultures.

**Figure 1:**
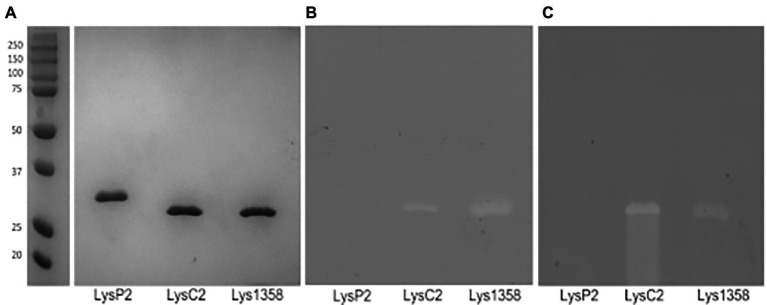
SDS-PAGE analysis of purified His-tagged LysP2, LysC2 and Lys1358 proteins **(A)** and zymograms prepared with *Lactococcus cremoris* NZ9000 **(B)** and *Micrococcus luteus* cells **(C)**. Precision Plus Protein™ All Blue Standards were used as a marker (lane 1).

Protein bands of the expected sizes were detected in 10% SDS-PAGE gels (Figure 1A). In addition, their PG hydrolytic activity was also revealed by zymography. LysC2 and Lys1358 produced clear lytic bands in zymograms prepared with *L. cremoris* NZ9000 (Figure 1B) and *Micrococcus luteus* cells (Figure 1C), whereas for LysP2 we were unable to detect any lytic activity regardless of the substrate cells. Because the renaturing conditions, e.g., pH and cations, may be critical to detect the bacteriolytic activity (Lepeuple et al., 1998), other renaturation buffers such as 50 mM MES (pH 6.0) and 3 M sodium acetate (pH 5.2) with or without 10 mM CaCl_2_, were tested but failed to reveal LysP2 activity. Hence, the following experiments were carried out with LysC2 and Lys1358.

### Exolytic activity of Lys1358 and LysC2 endolysins on CES and PG lactococcal mutants

**Figure 2:**
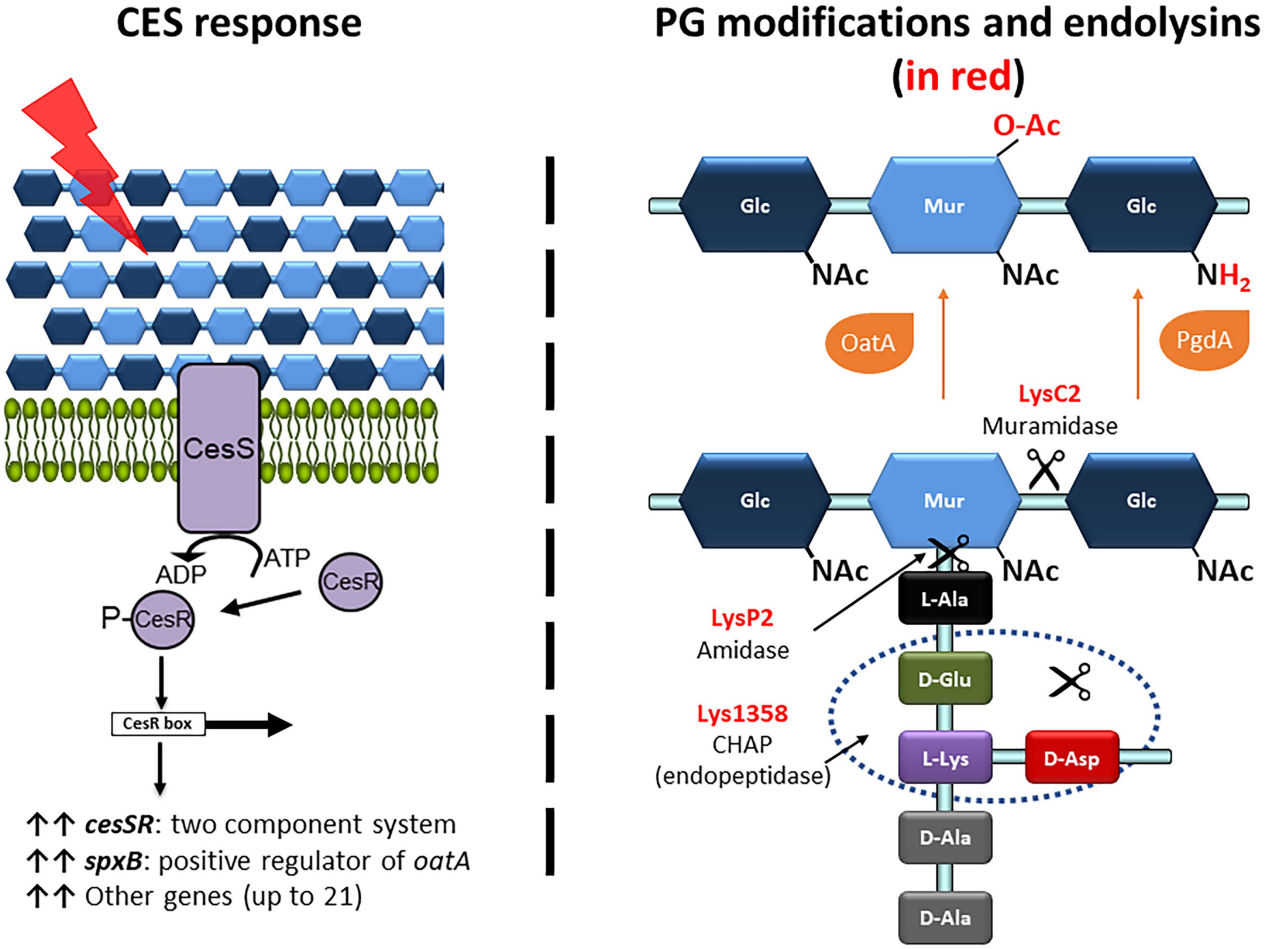
Overview of the CES response, the PG modifications and the PG bonds targeted by the lactococcal phage endolysins used in this work. (Right panel) The CES response is triggered upon damage (red ray) of the cell envelope that is sensed by the two-component system CesSR (purple proteins). The phosphorylated response regulator CesR binds to the CesR box and induces transcription of several genes, including *cesSR* and *spxB* (upright black arrows). SpxB, through binding to the RNA polymerase RpoA, drives transcription of *oatA* (not shown). (Left panel) The PG modifications of the N-acetyl-muramic acid (NAM) and N-acetyl-glucosamine (NAG) are shown in red. C_6_-*O*-acetylation and de-*N*-acetylation are mediated by OatA and PgdA, respectively (orange proteins). The bonds hydrolyzed by the endolysins LysC2, LysP2 and Lys1358, according to their conserved catalytic domains, are pointed by the scissors.

To evaluate the impact on the exolytic activity of modifications in the PG or an altered CES response, two sets of available mutants were collected in either *L. cremoris* MG1363 or *L. cremoris* NZ9000 background (see Table 1). For clarity, plus and minus signs stand for mutants overexpressing or lacking a particular gene(s). So-called CES mutants were those overexpressing genes involved in the CES response including the TCS genes *cesSR* (cesSR+) or its main effector *spxB* (spxB+). The other group, PG mutants, comprised those in genes that code for the PG modifying enzymes N-acetylglucosamine deacetylase PgdA and the O-acetyl transferase OatA. An overview of the CES response and the changes in the PG composition introduced by the different enzymes is shown in Figure 2. In general, these mutants embody a different degree of PG acetylation of both NAM and NAG residues.

**Figure 3:**
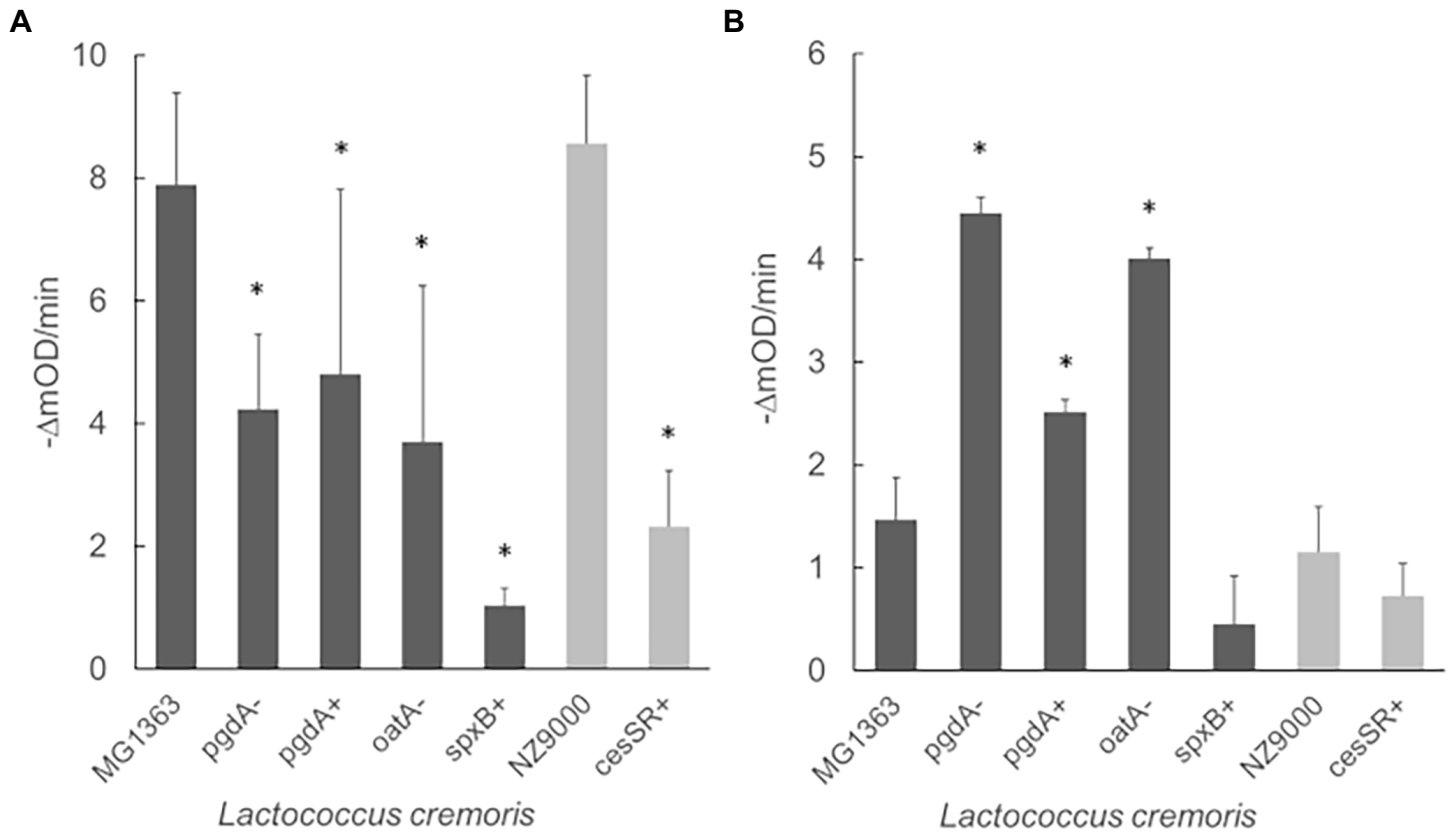
Exolytic activity of Lys1358 **(A)** and LysC2 **(B)** against CES and PG lactococcal mutants as determined by turbidity reduction assays. Purified Lys1358 (0.5 μM) and LysC2 (4.5 μM) were incubated with frozen cells and the decrease in OD_600_ (expressed in mili OD units, mOD) was monitored (-ΔmOD/min). Dark grey columns: *L. cremoris* MG1363 and derived mutants. Light grey columns: *L. cremoris* NZ9000 and derived mutants. *, Significantly different (*p* < 0.05) compared to the wild type *L. cremoris* MG1363 and NZ9000 cells.

The exolytic activity of Lys1358 and LysC2 was tested in turbidity reduction assays, following the decrease in the optical density (OD_600_) of cell suspensions. As shown in Figure 3, the activity of Lys1358 (CHAP catalytic domain) at 0.5 μM was mostly compromised against *L. cremoris* CES mutants cesSR+ and spxB+ where the lytic activity was only 27 and 13% of that on wild type cells, respectively (Figure 3A). Lys1358 activity on the PG mutants (pgdA-, pgdA+ and oatA-) was also diminished, albeit to a lesser extent, and no differences were observed when the substrate cells exhibit opposite degrees of NAG de-acetylation (pgdA+/pgdA-). Hence, Lys1358 appears to be more sensitive to an activated CES response and to a higher degree of NAM *O-*acetylation.

LysC2 (lysozyme catalytic domain) turned out to be less active against wild type cells than Lys1358 and cell lysis could only be observed at 4.5 μM, the highest concentration possible in our assay. Despite of this, differences in its exolytic activity on PG and CES mutants was noticed (Figure 3B). Contrary to Lys1358, LysC2 showed enhanced lytic activity on the PG mutants, mainly pgdA- and oatA-, increasing up to twofold, while on pgdA+, the activity was reduced compared to pgdA-. LysC2 was also less active against the CES mutants, although the difference was not statistically significant (*p* > 0.05). Thereby, LysC2 is more at the mercy of the degree of PG acetylation, in line with its muramidase specificity.

### Phage growth parameters on CES and PG lactococcal mutants

The results described above showed that the exolytic activity of phage endolysins may be either reduced or enhanced as function of the CES response and PG modifications. Hereinafter, we conducted experiments to evaluate their impact on several phage growth parameters. Since phage 1358 does not infect the reference strains *L. cremoris* MG1363 and NZ9000, the experiments were carried out with the virulent phage c2.

#### Phage infection experiments

The ability of phage c2 to propagate on the different mutants was assessed both in broth and solid medium. In these and the following experiments, a mutant devoid of the TCS CesSR (cesSR-) (Pinto et al., 2011) was also included. This mutant would be unable to turn on the TCS upon phage infection and would help us to fully appreciate the role of the CES response in phage growth.

To compare phage infection in broth, exponentially growing cells were challenged with phage c2 at a multiplicity of infection (MOI) of 0.2, and growth was followed until lysis had occurred, roughly up to 140 min post-infection. Following the method proposed by Xie et al. (2018), growth curves were integrated to determine the area under the curve and transformed into a single value, representing the differences in growth between the infected and the non-infected cultures. The lower the score, the poorer the performance of the phage on a particular mutant. This value (lytic score, Table 3) was normalized according to the reference strains *L. cremoris* MG1363 and NZ9000 to compare the lytic activity of phage c2 on the lactococcal mutants. The lytic scores were significantly reduced by half (*p* < 0.05) on spxB+ and by approximately 20% in cesSR+ and pgdA+. Noteworthy, the lytic score was restored on cesSR-, while there were no significant differences on phage performance on pgdA− and oatA− mutants (Table 3). Worth mentioning is that lower lytic scores were recorded in mutants that grow slower than their wild type counterparts. Therefore, the lower phage performance could be attributed, at least in part, to the physiology of the host rather than to their CW composition and its impact on the exolytic activity of LysC2.

**Table 3:** Phage c2 growth parameters during infection of lactococcal CES and PG mutants.

*Lactococcus cremoris*	Host growth rate (h^−1^)	Lytic score (%)	EOP	Adsorption (%)	Burst size (PFU)	Burst time (min)	Latent period (min)
NZ9000	0.49 ± 0.01	100	1	87.7 ± 0.7	154 ± 37	30	15
cesSR+	0.39 ± 0.01*	80.9 ± 3.5*	0.81 ± 0.08*	73.3 ± 2.7*	75 ± 7*	25	15
cesSR-	0.50 ± 0.00	101.8 ± 1.0	1.12 ± 0.07*	84.3 ± 3.1	149 ± 29	30	15
MG1363	0.50 ± 0.01	100	1	84.3 ± 2.5	129 ± 23	30	15
spxB+	0.23 ± 0.01*	53.3 ± 2.4*	0.85 ± 0.09*	74.0 ± 3.2*	153 ± 26	25	15
pgdA+	0.34 ± 0.00*	84.5 ± 4.9*	1.06 ± 0.11	60 ± 8.7*	128 ± 7	25	15
pgdA-	0.40 ± 0.00*	87.1 ± 12.2	0.93 ± 0.10	82.4 ± 3.6	99 ± 16	25	15
oatA-	0.52 ± 0.00	106.7 ± 3.4	0.98 ± 0.08	82.7 ± 8.0	112 ± 16	20	10

*Significantly different (*p* < 0.05) compare to the wild type *L. cremoris* NZ9000 and MG1363.

**Figure 4:**
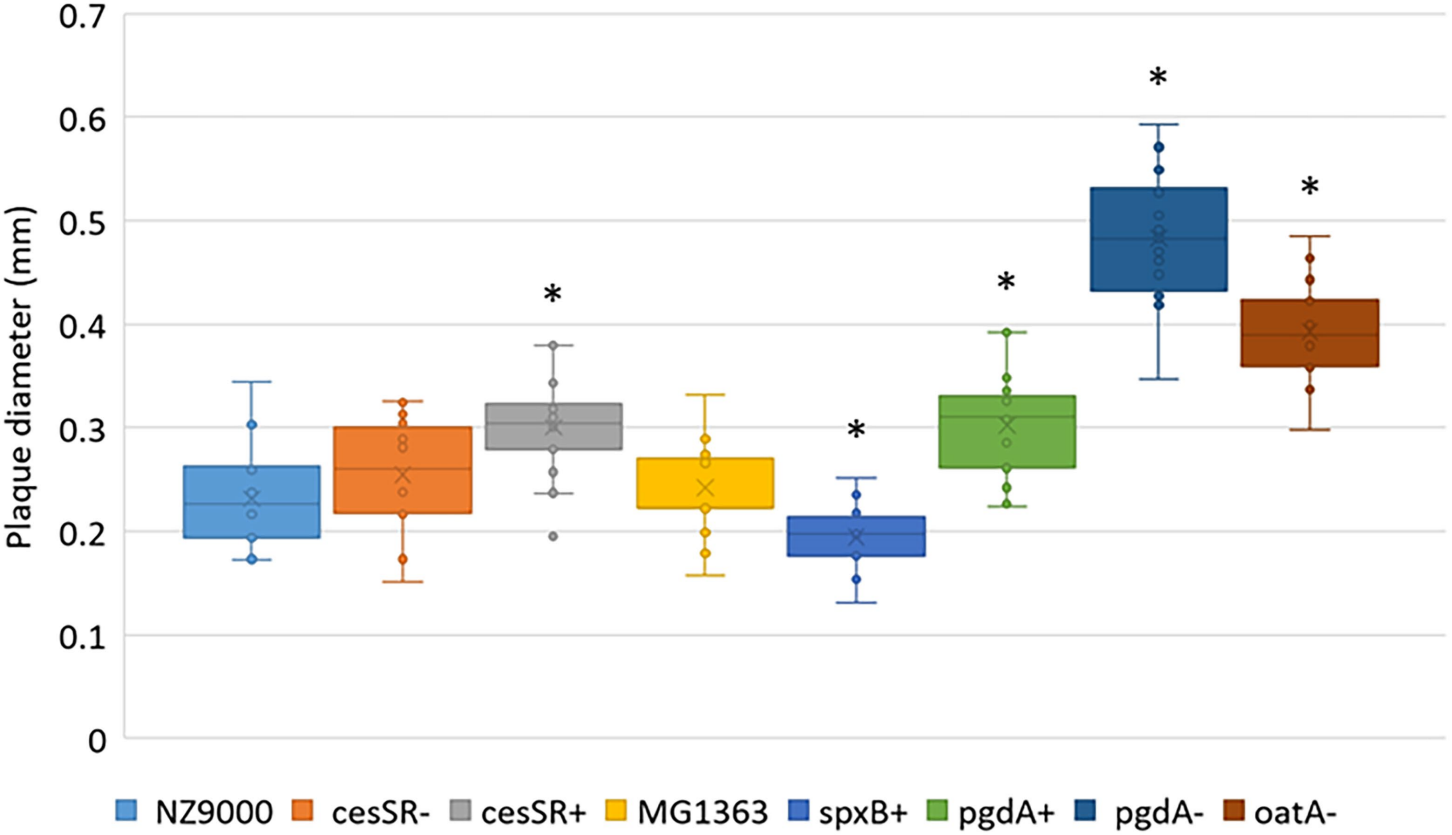
Average plaque diameter of phage c2 on lactococcal CES and PG mutants. *Significantly different (*p* < 0.05) compared to the wild type *L. cremoris* MG1363 and NZ9000.

The ability of phage c2 to propagate on the CES and PG mutants was further confirmed on solid medium. Phage c2 formed plaques on all the mutants, although the efficiency of plaquing (EOP) was again slightly lower on cesSR+ (0.81, *p* < 0.05) and spxB+ (0.85, *p* < 0.05; Table 3). The two CES mutants cesSR+ and cesSR- showed opposite trends, reinforcing the suggested defense role of this TCS against phage infection (Fallico et al., 2011). Remarkably, the plaque size of phage c2 varied depending on the mutants (Figure 4). The largest plaques were formed on those mutants susceptible to lysozyme (pgdA− and oatA−, see Table 1) with increments of 100 and 60%, respectively, compare to the wild type cells. Larger plaques were also noted on cesSR+ and pgdA+, although to a lesser extent, while the smallest c2 plaques were on spxB+, roughly 20% smaller than on the wild type host.

#### Adsorption and one-step growth curves

To get a deeper insight into the phage infection cycle in each of the lactococcal mutants, adsorption experiments and one-step curves were performed (Table 3). For phage c2, reversible adsorption occurs through interactions with an unidentified saccharide component of the CW and later, irreversible binding to the phage infection protein Pip followed by DNA ejection (Monteville et al., 1994). Under our experimental conditions, total adsorption was not largely affected, although lower adsorption values (*p* < 0.05) were recorded for cesSR+, spxB+ and pgdA+, the mutants with the lower lytic scores in infections in broth (Table 3).

The results of the one-step growth curves revealed that phage c2 behaved very much alike regardless the host. The same latent period (15 min) was recorded in all lactococcal mutants but oatA-, the later with a latent period 5 min shorter and the lowest burst time (Table 3). Burst sizes were also comparable (100–150 pfu per infected cell) with the exception of cesSR+ where the burst size was reduced by 50% (Table 3).

## Discussion

The bacterial CW is highly dynamic and can be either transiently modified in response to external cues or subjected to chemical modification through the activity of various enzymes. In this work, we asked the question if CW modifications could hinder the activity of endolysins from phages infecting dairy starter *Lactococcus,* so that phage propagation on these “endolysin-resistant” strains might be contained. Host lysis is a critical step for phage survival and previous reports had already shown that it can be targeted either by anti-phage systems such as AbiZ, that causes premature lysis of infected cells (Durmaz and Klaenhammer, 2007) or by host factors which are required for optimal lysis (São-José et al., 2000; Frías et al., 2009; Roces et al., 2013).

To identify such CW modifications, we initially opted to test the exolytic activity of three endolysins with different catalytic specificities (amidase, muramidase and CHAP) against a battery of lactococcal mutants, although turbidity reduction assays do not recapitulate fully the natural context of phage infection. Unfortunately, we were unable to find the right experimental conditions to measure the activity of LysP2 bearing an amidase_2 catalytic domain, one of the most abundant among lactococcal phages, according to a recent survey (Oechslin et al., 2022). The absence of LysP2 activity could be related to a putative multimeric nature of the enzyme. Whereas many endolysins produced by phages that infect Gram positive bacteria are monomeric, some require the assembly of additional domains which are synthesized from internal translational start sites within the endolysin gene, a situation more common than previously anticipated (Pinto et al., 2022). Inspection of the *E. coli* codon-optimized *lysP2* gene revealed the presence of an internal ATG start codon but the consensus ribosome binding site had been lost (see Supplementary Table S1). This absence could explain the lack of LysP2 activity, although this has not been experimentally demonstrated, and other factors (e.g., specific metal ion requirements, buffer composition or substrate specificity) cannot be disregarded.

The results from the turbidity assays showed that indeed modifications of the bacterial CW may compromise, but also improve, the exolytic activity of endolysins and underlined the difference and similarities within phage endolysins bearing CHAP (Lys1358) and muramidase (LysC2) catalytic domains. On one hand, the impact of the degree of PG acetylation (i.e., de-*N-*acetylation and C_6_-*O*-acetylation) had opposite effects on each endolysin. The exolytic activity of LysC2 was enhanced when tested on mutants such as those depleted of *pgdA* and *oatA*, known to be susceptible to egg-white lysozyme (Meyrand et al., 2007; Veiga et al., 2007). In contrast, Lys1358 was negatively affected. These two mutations modify defined PG sites which are specifically important for lysozyme activity (Bera et al., 2005) but other surface properties may also be altered. For example, anchoring of rhamnan is postulated to occur at the same site that is acetylated by OatA (Sadovskaya et al., 2017), anticipating an altered cell envelope configuration, which could be relevant for Lys1358 activity.

On the other hand, both endolysins showed a reduced exolytic activity on cesSR+ and spxB+, being susceptibility to Lys1358 lytic action more heavily reduced. This could be explained by a more broader modification of the cell envelope caused by these mutations. The TCS CesSR regulates up to 21 genes, supposedly involved in protecting cells from CW damage, and SpxB may compete with other Spx proteins, leading to so far unknown phenotypes (reviewed by Martínez et al., 2020). Therefore, it is plausible that other modifications are responsible for the altered exolytic activity of the two lactococcal phage endolysins. Moreover, such modifications may also influence endolysin binding to the substrate cells, accounting for their overall lytic activity, as observed when engineering cell wall binding domains (Schmelcher et al., 2011). We have previously reported reduced binding of the cell wall binding domain of LysTP712 to *L. lactis* mutants lacking *ftsH* (Roces et al., 2016).

As for the consequences for phage infection, no drastic impacts were seen on phage c2 in terms of resistance. Nonetheless, the two CES mutants cesSR+ and spxB+ tend to curb phage growth slightly, as shown by the lowest lytic scores and EOP values. However, it is not possible to directly infer that this is due to the reduced activity of its endolysin. Firstly, because these mutations did not strongly inhibit LysC2. Secondly, at least, other two factors could account for the lower phage fitness such as the lower growth rate of these mutants and the lower phage adsorption values. Both factors are known to influence phage infection dynamics (Hadas et al., 1997; Dennehy and Abedon, 2020). In the particular case of the cesSR+ mutant, the burst size was remarkably reduced. This may reflect the contribution of this TCS to overcome the stress caused upon phage infection, compromising phage replication and protein synthesis (Fallico et al., 2011). The results for the pgdA+ mutant also exemplify the difficulties encountered in stablishing a correlation between the exolytic activity and phage infection. In this case, in spite of the enhanced exolytic activity of LysC2, the lytic score is reduced, likely due to the lower phage adsorption values.

Mutations that enhanced the exolytic activity of LysC2 such as the lack of NAG de-*N*-acetylation (pgdA-) and PG *O*-acetylation (oatA-) did have an impact on phage growth on solid media. On these mutants, phage c2 forms considerably larger plaques (see Figure 4) which could be explained by the shorter latent period, at least, in the case of oatA-. In this scenario, phage virions would be released quicker increasing the number of replicative cycles, so more bacteria would become infected and, consequently, plaques are enlarged (Abedon and Culler, 2007; Liu et al., 2022).

In summary, this work has demonstrated that certain CW modifications have an impact on the exolytic activity of endolysins in a similar fashion as described for autolysins or innate immunity factors such as lysozyme. While in the context of milk fermentations, this may not have important implications, so it does when endolysins targeting pathogenic bacteria are proposed as antimicrobials to fight infections or as food biopreservatives (Schmelcher et al., 2012; Murray et al., 2021). Based on our data, limited to a single endolysin/phage pair, hindering the exolytic activity of endolysins does not appear to impose a major burden to phage growth.

## Data availability statement

The datasets presented in this study can be found in online repositories. The names of the repository/repositories and accession number(s) can be found below: Raw data are available at the institutional repository of the Spanish National Research Council, Digital.CSIC https://doi.org/10.20350/digitalCSIC/14766.

## Author contributions

AR and BM contributed to conception and design of the study. SE, MP, and CR performed the experiments. SE and BM carried out formal analysis and wrote the first draft of the manuscript. All authors contributed to manuscript revision, read, and approved the submitted version.

## Funding

This work has been funded by grants AYUD/2021/52120 (Program of Science, Technology and Innovation 2018–2022, Principado de Asturias, FICYT, FEDER-UE), grant BIO2017-88147-R (MCIN/AEI/10.13039/501100011033 and by “ERDF A way of making Europe”) and grant PID2020-119697RB-I00 (MCIN/AEI/10.13039/501100011033). CR is a fellow of the program “Ayudas Severo Ochoa” of the Principality of Asturias (BP20 006).

## Conflict of interest

The authors declare that the research was conducted in the absence of any commercial or financial relationships that could be construed as a potential conflict of interest.

## Publisher’s note

All claims expressed in this article are solely those of the authors and do not necessarily represent those of their affiliated organizations, or those of the publisher, the editors and the reviewers. Any product that may be evaluated in this article, or claim that may be made by its manufacturer, is not guaranteed or endorsed by the publisher.

## References

[ref1] Abedon S. T. Culler R. R. (2007). Optimizing bacteriophage plaque fecundity. J. Theor. Biol. 249, 582–592. doi: 10.1016/j.jtbi.2007.08.006, PMID: 17919662

[ref2] Bera A. Herbert S. Jakob A. Vollmer W. Götz F. (2005). Why are pathogenic staphylococci so lysozyme resistant? The peptidoglycan *O*-acetyltransferase OatA is the major determinant for lysozyme resistance of *Staphylococcus aureus*. Mol. Microbiol. 55, 778–787. doi: 10.1111/j.1365-2958.2004.04446.x, PMID: 15661003

[ref3] Bertozzi Silva J. Storms Z. Sauvageau D. (2016). Host receptors for bacteriophage adsorption. FEMS Microbiol. Lett. 363:fnw002. doi: 10.1093/femsle/fnw002 26755501

[ref4] Chapot-Chartier M. P. Kulakauskas S. (2014). Cell wall structure and function in lactic acid bacteria. Microb. Cell Factories 13:S9. doi: 10.1186/1475-2859-13-S1-S9, PMID: 25186919 PMC4155827

[ref5] Chmielewska-Jeznach M. Bardowski J. K. Szczepankowska A. K. (2020). *Lactococcus Ceduovirus* phages isolated from industrial dairy plants-from physiological to genomic analyses. Viruses 12:280. doi: 10.3390/v12030280, PMID: 32138347 PMC7150918

[ref6] Dennehy J. J. Abedon S. T. (2020). “Phage infection and lysis” in Bacteriophages. eds. Harper D. R. Abedon S. T. Burrowes B. H. McConville M. L. (Springer: Cham) doi: 10.1007/978-3-319-40598-8_53-1

[ref7] Durmaz E. Klaenhammer T. R. (2007). Abortive phage resistance mechanism AbiZ speeds the lysis clock to cause premature lysis of phage-infected *Lactococcus lactis*. J. Bacteriol. 189, 1417–1425. doi: 10.1128/JB.00904-06, PMID: 17012400 PMC1797342

[ref8] Escobedo S. Campelo A. B. Wegmann U. García P. Rodríguez A. Martínez B. (2019). Insight into the lytic functions of the lactococcal prophage TP712. Viruses 11:881. doi: 10.3390/v11100881, PMID: 31546996 PMC6832245

[ref9] Fallico V. Ross R. P. Fitzgerald G. F. McAuliffe O. (2011). Genetic response to bacteriophage infection in *Lactococcus lactis* reveals a four-strand approach involving induction of membrane stress proteins, D-alanylation of the cell wall, maintenance of proton motive force, and energy conservation. J. Virol. 85, 12032–12042. doi: 10.1128/JVI.00275-11, PMID: 21880765 PMC3209278

[ref10] Frías M. J. Melo-Cristino J. Ramírez M. (2009). The autolysin LytA contributes to efficient bacteriophage progeny release in *Streptococcus pneumoniae*. J. Bacteriol. 191, 5428–5440. doi: 10.1128/JB.00477-09, PMID: 19581370 PMC2725628

[ref11] Gasson M. J. (1983). Plasmid complements of *Streptococcus lactis* NCDO 712 and other lactic streptococci after protoplast-induced curing. J. Bacteriol. 154, 1–9. doi: 10.1128/jb.154.1.1-9.1983, PMID: 6403500 PMC217423

[ref12] Grishin A. V. Karyagina A. S. Vasina D. V. Vasina I. V. Gushchin V. A. Lunin V. G. (2020). Resistance to peptidoglycan-degrading enzymes. Crit. Rev. Microbiol. 46, 703–726. doi: 10.1080/1040841X.2020.1825333, PMID: 32985279

[ref13] Hadas H. Einav M. Fishov I. Zaritsky A. (1997). Bacteriophage T4 development depends on the physiology of its host *Escherichia coli*. Microbiology 143, 179–185. doi: 10.1099/00221287-143-1-179 9025292

[ref14] Jordan S. Hutchings M. I. Mascher T. (2008). Cell envelope stress response in gram-positive bacteria. FEMS Microbiol. Rev. 32, 107–146. doi: 10.1111/j.1574-6976.2007.00091.x 18173394

[ref15] Kuipers O. P. De Ruyter P. G. G. A. Kleerebezem M. De Vos W. M. (1998). Quorum sensing-controlled gene expression in lactic acid bacteria. J. Biotech. 64, 15–21. doi: 10.1016/S0168-1656(98)00100-X

[ref16] Latka A. Maciejewska B. Majkowska-Skrobek G. Briers Y. Drulis-Kawa Z. (2017). Bacteriophage-encoded virion-associated enzymes to overcome the carbohydrate barriers during the infection process. Appl. Microbiol. Biotechnol. 101, 3103–3119. doi: 10.1007/s00253-017-8224-6, PMID: 28337580 PMC5380687

[ref17] Lepeuple A. S. Van Gemert E. Chapot-Chartier M. P. (1998). Analysis of the bacteriolytic enzymes of the autolytic *Lactococcus lactis* subsp*. cremoris* strain AM2 by renaturing polyacrylamide gel electrophoresis: identification of a prophage-encoded enzyme. Appl. Environ. Microbiol. 64, 4142–4148. doi: 10.1128/AEM.64.11.4142-4148.1998, PMID: 9797258 PMC106620

[ref18] Liu C. Hong Q. Chang R. Y. K. Kwok P. C. L. Chan H. K. (2022). Phage-antibiotic therapy as a promising strategy to combat multidrug-resistant infections and to enhance antimicrobial efficiency. Antibiotics 11:570. doi: 10.3390/antibiotics11050570, PMID: 35625214 PMC9137994

[ref19] Madera C. García P. Janzen T. Rodríguez A. Suárez J. E. (2003). Characterization of technologically proficient wild *Lactococcus lactis* strains resistant to phage infection. Int. J. Food Microbiol. 86, 213–222. doi: 10.1016/s0168-1605(03)00042-4, PMID: 12915032

[ref20] Mahony J. Cambillau C. van Sinderen D. (2017). Host recognition by lactic acid bacterial phages. FEMS Microbiol. Rev. 41, S16–S26. doi: 10.1093/femsre/fux019, PMID: 28830088

[ref21] Marcó M. B. Moineau S. Quiberoni A. (2012). Bacteriophages and dairy fermentations. Bacteriophage 2, 149–158. doi: 10.4161/bact.21868 23275866 PMC3530524

[ref22] Martínez B. Rodríguez A. Kulakauskas S. Chapot-Chartier M. P. (2020). Cell wall homeostasis in lactic acid bacteria: threats and defences. FEMS Microbiol. Rev. 44, 538–564. doi: 10.1093/femsre/fuaa021, PMID: 32495833 PMC7476776

[ref23] Martínez B. Zomer A. L. Rodríguez A. Kok J. Kuipers O. P. (2007). Cell envelope stress induced by the bacteriocin Lcn972 is sensed by the Lactococcal two-component system CesSR. Mol. Microbiol. 64, 473–486. doi: 10.1111/j.1365-2958.2007.05668.x, PMID: 17493129

[ref24] Meyrand M. Boughammoura A. Courtin P. Mézange C. Guillot A. Chapot-Chartier M. P. (2007). Peptidoglycan N-acetylglucosamine deacetylation decreases autolysis in *Lactococcus lactis*. Microbiology 153, 3275–3285. doi: 10.1099/mic.0.2007/005835-0, PMID: 17906127

[ref25] Moak M. Molineux I. J. (2004). Peptidoglycan hydrolytic activities associated with bacteriophage virions. Mol. Microbiol. 51, 1169–1183. doi: 10.1046/j.1365-2958.2003.03894.x, PMID: 14763988

[ref26] Moineau S. Durmaz E. Pandian S. Klaenhammer T. R. (1993). Differentiation of two abortive mechanisms by using monoclonal antibodies directed toward lactococcal bacteriophage capsid proteins. Appl. Environ. Microbiol. 59, 208–212. doi: 10.1128/aem.59.1.208-212.1993, PMID: 16348844 PMC202079

[ref27] Monteville M. R. Ardestani B. Geller B. L. (1994). Lactococcal bacteriophages require a host cell wall carbohydrate and a plasma membrane protein for adsorption and ejection of DNA. Appl. Environ. Microbiol. 60, 3204–3211. doi: 10.1128/aem.60.9.3204-3211.1994, PMID: 16349376 PMC201790

[ref29] Murray E. Draper L. A. Ross R. P. Hill C. (2021). The advantages and challenges of using endolysins in a clinical setting. Viruses 13:680. doi: 10.3390/v13040680, PMID: 33920965 PMC8071259

[ref30] Oechslin F. Zhu X. Dion M. B. Shi R. Moineau S. (2022). Phage endolysins are adapted to specific hosts and are evolutionarily dynamic. PLoS Biol. 20:e3001740. doi: 10.1371/journal.pbio.3001740, PMID: 35913996 PMC9371310

[ref31] Oliveira H. Melo L. D. Santos S. B. Nóbrega F. L. Ferreira E. C. Cerca N.. (2013). Molecular aspects and comparative genomics of bacteriophage endolysins. J. Virol. 87, 4558–4570. doi: 10.1128/JVI.03277-12, PMID: 23408602 PMC3624390

[ref32] Pillidge C. J. Jarvis A. W. (1988). DNA restriction maps and classification of the lactococcal bacteriophages c2 and skl. J. Dairy. Sci. Technol. 23, 411–416.

[ref33] Pinto D. Gonçalo R. Louro M. Silva M. S. Hernandez G. Cordeiro T. N.. (2022). On the occurrence and multimerization of two-polypeptide phage endolysins encoded in single genes. Microbiol. Spectr. 10:e0103722. doi: 10.1128/spectrum.01037-22, PMID: 35876588 PMC9430671

[ref34] Pinto J. P. Kuipers O. P. Marreddy R. K. Poolman B. Kok J. (2011). Efficient overproduction of membrane proteins in *Lactococcus lactis* requires the cell envelope stress sensor/regulator couple CesSR. PLoS One 6:e21873. doi: 10.1371/journal.pone.0021873, PMID: 21818275 PMC3139573

[ref35] Roces C. Campelo A. B. Escobedo S. Wegmann U. García P. Rodríguez A.. (2016). Reduced binding of the endolysin LysTP712 to *Lactococcus lactis* ΔftsH contributes to phage resistance. Front. Microbiol. 7:138. doi: 10.3389/fmicb.2016.00138, PMID: 26904011 PMC4749879

[ref36] Roces C. Wegmann U. Campelo A. B. García P. Rodríguez A. Martínez B. (2013). Lack of the host membrane protease FtsH hinders release of the *Lactococcus lactis* bacteriophage TP712. J. Gen. Virol. 94, 2814–2818. doi: 10.1099/vir.0.057182-0, PMID: 24018314

[ref001] Sadovskaya I. Vinogradov E. Courtin P. Armalyte J. Meyrand M. Giaouris E.. (2017). Another brick in the wall: a rhamnan polysaccharide trapped inside peptidoglycan of *Lactococcus lactis*. mBio 8:e01303-17. doi: 10.1128/mBio.01303-17 28900021 PMC5596347

[ref37] Sambrook J. Fritsch E. R. Maniatis T. (1989). Molecular Cloning: A Laboratory Manual, 2nd Edn Cold Spring Harbor, NY: Cold Spring Harbor Laboratory Press.

[ref38] São-José C. Parreira R. Vieira G. Santos M. A. (2000). The N-terminal region of the *Oenococcus oeni* bacteriophage fOg44 lysin behaves as a bona fide signal peptide in *Escherichia coli* and as a cis-inhibitory element, preventing lytic activity on oenococcal cells. J. Bacteriol. 182, 5823–5831. doi: 10.1128/JB.182.20.5823-5831.2000, PMID: 11004183 PMC94706

[ref39] Schmelcher M. Donovan D. M. Loessner M. J. (2012). Bacteriophage endolysins as novel antimicrobials. Future Microbiol. 7, 1147–1171. doi: 10.2217/fmb.12.97, PMID: 23030422 PMC3563964

[ref40] Schmelcher M. Tchang V. S. Loessner M. J. (2011). Domain shuffling and module engineering of listeria phage endolysins for enhanced lytic activity and binding affinity. Microb. Biotechnol. 4, 651–662. doi: 10.1111/j.1751-7915.2011.00263.x, PMID: 21535426 PMC3819014

[ref41] Steen A. Buist G. Kramer N. E. Jalving R. Benus G. F. Venema G.. (2008). Reduced lysis upon growth of *Lactococcus lactis* on galactose is a consequence of decreased binding of the autolysin AcmA. Appl. Environ. Microbiol. 74, 4671–4679. doi: 10.1128/AEM.00103-08, PMID: 18539791 PMC2519321

[ref42] Veiga P. Bulbarela-Sampieri C. Furlan S. Maisons A. Chapot-Chartier M. P. Erkelenz M.. (2007). SpxB regulates O-acetylation-dependent resistance of *Lactococcus lactis* peptidoglycan to hydrolysis. J. Biol. Chem. 282, 19342–19354. doi: 10.1074/jbc.M611308200, PMID: 17485463

[ref43] Veiga P. Erkelenz M. Bernard E. Courtin P. Kulakauskas S. Chapot-Chartier M. P. (2009). Identification of the asparagine synthase responsible for D-Asp amidation in the *Lactococcus lactis* peptidoglycan interpeptide crossbridge. J. Bacteriol. 191, 3752–3757. doi: 10.1128/JB.00126-09, PMID: 19329637 PMC2681893

[ref44] Viscardi M. Capparelli R. Di Matteo R. Carminati D. Giraffa G. Iannelli D. (2003). Selection of bacteriophage-resistant mutants of *Streptococcus thermophilus*. J. Microbiol. Methods 55, 109–119. doi: 10.1016/s0167-7012(03)00146-5, PMID: 14500002

[ref45] Xie Y. Wahab L. Gill J. J. (2018). Development and validation of a microtiter plate-based assay for determination of bacteriophage host range and virulence. Viruses 10:189. doi: 10.3390/v10040189, PMID: 29649135 PMC5923483

