# Supplementary material for: Cell wall modifications that alter the exolytic activity of lactococcal phage endolysins have little impact on phage growth
Source: Front Microbiol. 2023 Jan 20;14:1106049. doi: 10.3389/fmicb.2023.1106049 (PMC9894900; doi:10.3389/fmicb.2023.1106049)
Supplement: Supplementary file 1 [file Data_Sheet_1.PDF]

Table S1. Original and optimized nucleotide sequences of the phage endolysins used in this work. In red, putative internal ATG codons and ribosome binding sites underlined according to the preliminary prediction reported by Pinto et al. 2022 DOI: https://doi.org/10.1128/spectrum.01037-22

> Lactococcus lactis phage C2 endolysin. Lys_C2

```
  1 ATGAAAGTAT CACAAAACGG TTTGAACTTG ATTAAAGAGT TTGAGGGTTG TAGGTTGACT
 61 GCTTATAAAC CTGTACCGTG GGAACAAATG TACACTATCG GTTGGGGTCA TTATGGAGTC
121 ACAGCAGGTA CAACTTGGAC ACAAGCACAA GCTGATAGTC AGCTAGAGAT TGACATCAAT
181 AATAAGTATG CACCTATGGT TGACGCTTAC GTAAAAGGCA AAGCAAACCA AAACGAGTTT
241 GACGCCTTAG TGTCATTGGC TTATAACTGT GGTAATGTTT TCGTTGCTGA CGGTTGGGCG
301 CCATTCAGTC ATGCTTATTG TGCTTCAATG ATTCCGAAGT ATCGTAATGC AGGCGGTCAA
361 GTCTTACAAG GCTTAGTAAG ACGCAGACAG GCAGAGCTTA ACTTATTTAA TAAACCAGTA
421 TCAAGTAATT CAAACCAAAA CAATCAAACA GGAGGAATGA TAAAAATGTA CCTTATTATA
481 GGACTAGATA ATTCAGGTAA AGCTAAACAT TGGTATGTTT CTGACGGTGT AAGTGTTCGT
541 CATGTTCGTA CAATTCGTAT GTTGGAAAAC TATCAAAACA AATGGGCTAA ACTTAACTTG
601 CCAGTTGATA CAATGTTTAT TGCAGAAATC GAAGCAGAGT TTGGACGTAA GATTGACATG
661 GCTTCAGGAG AAGTGAAATA G
```

>lys_C2 optimized

```
  1 ATGAAGGTGT CGCAGAATGG GTTAAATCTG ATCAAGGAAT TCGAAGGATG CCGTCTGACC
 61 GCGTACAAGC CGGTGCCTTG GGAGCAGATG TATACGATTG GCTGGGGCCA CTACGGCGTT
121 ACCGCCGGCA CGACATGGAC TCAGGCTCAG GCCGACTCAC AACTGGAAAT CGATATTAAC
181 AACAAATACG CGCCGATGGT AGATGCATAT GTTAAGGGTA AGGCGAATCA GAATGAATTC
241 GATGCGCTGG TAAGTCTGGC ATACAATTGC GGCAACGTCT TTGTGGCGGA TGGATGGGCA
301 CCGTTTTCAC ACGCGTACTG CGCGAGCATG ATCCCTAAAT ACCGCAACGC TGGCGGCCAG
361 GTGCTGCAGG GGCTGGTGCG CCGTCGTCAA GCGGAATTAA ATTTGTTCAA CAAGCCTGTG
421 TCGAGCAACA GTAATCAGAA TAACCAGACC GGTGGTATGA TTAAGATGTA TTTGATCATT
481 GGGCTTGACA ACAGCGGCAA GGCGAAGCAC TGGTACGTGA GCGATGGCGT GAGCGTGCGC
541 CACGTGCGCA CCATCCGCAT GCTTGAGAAT TACCAGAATA AGTGGGCAAA GTTGAATCTG
601 CCGGTGGACA CGATGTTCAT CGCTGAGATT GAGGCGGAAT TCGGCCGCAA AATCGATATG
661 GCGAGTGGCG AGGTCAAG
```

> Lactococcus lactis phage P2 endolysin

```
  1 ATGAATATAA CTAATGCTGG CGTTCGTGGG TATAATCCTA CCGGGGTTGT AATCCACAAT
 61 GATGCTGGGT CAAATGGTGC TAACGCCAGT TTCTATGATG ATTGGCTACC TAAGCAAAAC
121 CCAGAAAATG GCTTTGCTCA TGTTTATATT GGAAACGACG GAAGATTGCA GGCTTCTGAC
181 TTCTCTAACA TGGCATGGCA TTGTGCTAAC TCATACGGTA ATGCAAATTA TGCCAGTTGG
241 GAAGTATGCC AATCAGAGGG CGATTTAAAT CAGTTCTTGA GAAATGAGCA AGCGGTACTA
301 GATGATGTAG CTAAGTACAT GAAACAATGG GGGCTAACTC CTAATCGTGA TACTGTGAAG
361 CTACATCAGG AGTTATCATC TACTTCATGC CCTAGACGTT CAGTAGAAGC TCACGGTGGC
421 ACGGTAGAGA GTTGTCGCTC ATACTTTATC ACAGAACTAA ACAAGCGCCT TACAGGACAA
481 ACTAGTGGCA CAGTCGCAGT AAACAATACA CAAACAAATA CAGAATTAGA GGACGACGAT
541 TTAATGAAAT TTACATATAC AAATGGCGAT AAAACAACTT ACTACTTTAA TGGCGAAAAA
601 GTTATCGCTC TATCACACCC AGACCAATTG GCAATTGTTC GTAAAACTTA TAAAGAAACA
661 ACTGGCAAAG ACCTTAAAAA CTTCGATTGG AAAGGTTCGC CTATTGATAT TCGTTTCATG
721 CAAGCTAACG GAATCGACAA ACCAATCATT GCTAAAAAAT AA
```

>Lys_P2 optimized sequence

```
  1 ATGAACATTA CGAATGCAGG GGTGCGCGGC TATAACCCGA CGGGTGTGGT TATTCATAAC
 61 GACGCGGGTA GCAACGGCGC AAATGCAAGC TTTTACGACG ACTGGCTGCC CAAACAGAAT
121 CCGGAGAACG GTTTCGCGCA CGTGTACATC GGGAATGATG GTCGTTTACA AGCCTCAGAT
181 TTCTCGAATA TGGCCTGGCA CTGCGCGAAT TCGTATGGGA ACGCGAACTA CGCAAGCTGG
241 GAAGTCTGTC AGTCCGAAGG TGACCTTAAC CAATTTCTGC GTAACGAACA GGCCGTTTTA
301 GACGACGTTG CGAAATATAT GAAGCAGTGG GGTTTAACCC CCAACCGCGA CACGGTAAAA
361 CTGCACCAAG AACTGAGCTC GACCAGCTGT CCGCGTCGCA GTGTGGAAGC ACATGGCGGT
421 ACTGTGGAAT CCTGCCGTAG CTATTTCATT ACCGAGCTGA ATAAACGTTT GACCGGCCAG
481 ACATCCGGAA CCGTGGCCGT GAATAACACC CAGACGAACA CTGAGCTCGA AGATGATGAC
541 CTCATGAAGT TCACTTACAC CAACGGAGAC AAGACCACCT ATTATTTCAA CGGGGAGAAG
601 GTCATTGCCC TCTCGCATCC GGATCAGCTG GCGATCGTGC GCAAGACCTA CAAGGAGACG
661 ACAGGAAAGG ATCTGAAGAA TTTTGACTGG AAGGGGTCCC CCATCGACAT CCGCTTTATG
721 CAGGCCAATG GGATTGATAA GCCCATTATC GCCAAGAAG
```

>Lactococcus lactis phage 1358 endolysin. Lys_1358

```
  1 ATGGTAAATC AAGCACAGGT CAAGCAATGG ATTGACACAC ACGTCGGCAA ATGGGTCGAC
 61 TTCGACGGCA TGTACGGCGC GCAATGCATG GACTTAGCCG TCCAATATGC ACACGACCTT
121 TGGGGCTTTC GCCTTACAGG TAACGCCGAA AACTTGCGAA ATCAAGCTTT GCCCGCAGGG
181 TGGCAACGCA TTAAGAATTC GCGCGGCTTC GTTCCACAGC AGGGCGACAT CTTCGTGTGG
241 TACGGCACTA AGCACCCGTA CGGGCACACA GGTATCGTCA TCAGCGCGAC GTTGAACAAC
301 TACACCGCGC TCGAGCAAAA CATGGTTACA GGTAAAGGAC AAGCCGCTGA CAAAGCGGCA
361 ATCAACACAC GCCCTTATCC AAGCGAATTT TGGGGCGTTG TGCGCCCGCC GATTACTTCG
421 GGGGGAAATA TTACCCCACC CGACCAAAAC GGCACAGGCG GCAAACTGAC GGCACAGCGG
481 GGTACATTCA AGGCAAACAG CGCCGTGAAT ATCCGCCGCG CACCGAATAC AAAAACAGGA
541 ACAGTCGCAG GCGTCCTCAA AGCAGGTCAG ACCGTGAACT ATGATAATAT CATTGACGCG
601 GACGGCTGGC GTTGGGTTTC ATGGGTCGGC GCTTCGGGCA ACCGAAATTA TAGCGCAGTC
661 CGCCGCTTGT CTGACAATTT CCGAACAGGG TTGTGCTATT AA
```

>Lys_1358 optimized sequence

```
  1 ATGGTTAACC AGGCCCAAGT TAAACAGTGG ATCGATACCC ATGTAGGAAA GTGGGTTGAT
 61 TTTGATGGTA TGTATGGAGC TCAGTGTATG GATCTGGCAG TACAGTACGC GCATGATTTA
121 TGGGGTTTCC GTCTGACCGG CAATGCGGAG AATCTGCGCA ACCAGGCGCT GCCGGCTGGC
181 TGGCAGCGTA TCAAGAACAG CCGTGGGTTT GTGCCTCAAC AAGGAGATAT TTTCGTCTGG
241 TATGGTACCA AACATCCTTA TGGACATACC GGCATTGTGA TTAGTGCCAC CCTGAATAAT
301 TATACAGCTT TAGAACAGAA TATGGTAACC GGCAAGGGCC AGGCGGCAGA TAAGGCCGCC
361 ATTAATACGC GTCCCTACCC GTCTGAGTTC TGGGGTGTGG TACGTCCGCC AATCACCTCT
421 GGTGGCAACA TCACACCGCC GGATCAGAAT GGGACTGGTG GTAAGTTAAC CGCCCAACGT
481 GGCACCTTTA AAGCCAATTC GGCTGTTAAC ATTCGTCGTG CGCCCAACAC CAAGACCGGT
541 ACCGTTGCTG GTGTACTTAA GGCGGGCCAA ACAGTTAATT ACGACAACAT TATCGATGCC
601 GATGGTTGGC GCTGGGTAAG CTGGGTGGGT GCCAGTGGTA ATCGCAACTA CTCTGCGGTA
661 CGTCGTCTGT CAGATAACTT TCGCACTGGT TTATGTTAC
```
